# Hyper-responsiveness to warfarin in a young patient with the VKORC1 -1639GA/CYP2C9*1*46 genotype: a case report

**DOI:** 10.1186/s12959-022-00425-8

**Published:** 2022-10-27

**Authors:** Weam Aldiban, Yara Altawil, Samir Hussein, Majd Aljamali, Lama A. Youssef

**Affiliations:** 1 Program of Clinical and Hospital Pharmacy, Department of Pharmaceutics and Pharmaceutical Technology, Faculty of Pharmacy, Damascus University, Damascus, Syrian Arab Republic; 2 Faculty of Pharmacy, International University for Science and Technology (IUST), Daraa, Syrian Arab Republic; 3 AL Basel Hospital, Homs, Syrian Arab Republic; 4 Department of Biochemistry and Microbiology, Faculty of Pharmacy, Damascus University, Damascus, Syrian Arab Republic; 5 National Commission for Biotechnology, Damascus, Syrian Arab Republic

**Keywords:** Hyper-responsiveness, Warfarin, INR, CYP2C9, VKORC1, Polymorphism

## Abstract

**Background:**

Warfarin is the most widely used oral anticoagulant; nevertheless, dosing of warfarin is problematic for clinicians worldwide. Inter-individual variability in response to warfarin is attributed to genetic as well as non-genetic factors. Pharmacogenomics studies have identified variants in CYP2C9 and VKORC1 genes as significant predictors of warfarin dose, however, phenotypes of rare variants are not well characterized.

**Case presentation:**

We report a case of hyper-responsiveness to warfarin in a 22-year-old outpatient with Crohn's disease who presented with a swollen, red, and painful left calf. Deep venous thrombosis (DVT) in the left lower extremity was confirmed via ultrasonography, and hence, anticoagulation therapy of heparin and concomitant warfarin was initiated. Warfarin dose of 7.5 mg/day was estimated by the physician based on clinical factors. Higher than the expected international normalized ratio (INR) value of 4.5 necessitated the reduction of the warfarin dose to 5 and eventually to 2.5 mg/day to reach a therapeutic INR value of 2.6. Pharmacogenetic profiling of the VKORC1 **-**1639G** > **A and CYP2C9 *2, *3, *4, *5, *8, *14, *20, *24, *26, *33, *40, *41, *42, *43, *45, *46, *55, *62, *63, *66, *68, *72, *73 and *78 revealed a VKORC1-1639GA/CYP2C9*1*46 genotype. The lower catalytic activity of the CYP2C9*46 (A149T) variant was previously reported in in vitro settings.

**Conclusions:**

This is the first report on a case of warfarin hyper-responsive phenotype of a patient with the heterozygous CYP2C9*1*46 polymorphism.

## Background

Since its emergence, personalized medicine (PM) has placed itself as an advanced practice of medicine that uses individuals' non-genetic and genetic profiles to guide and support the decision-making processes regarding prevention, diagnosis, and treatment of disease. Pharmacogenetics and pharmacogenomics (PGx) have been widely recognized as pillars of PM [1].

Warfarin is a classic example of the clinical utility of PGx- guided personalized medicine [2]. On one hand, warfarin is the most commonly prescribed oral anticoagulant in the world, with indications spanning a wide array of thromboembolic disorders, including prevention and treatment of DVT and pulmonary embolism, in addition to atrial fibrillation and prosthetic heart valves [3]. On the other hand, potential warfarin-drug interactions with a multitude of drugs and foods along with warfarin’s narrow therapeutic index raise serious safety concerns, with bleeding being the most serious and most frequent adverse effect of warfarin [3]. Moreover, inter-individual variations in dose requirements that ensure the stability of anticoagulation and minimal bleeding risk make warfarin dosing a challenging task [4].

A value within the INR's range of [2.0–3.0] is a practical indicator of the safety and effectiveness of warfarin [5]. Environmental factors influencing warfarin's benefit-harm profile encompass age, gender, body weight, concurrent medications, diet (i.e., intake of vitamin K), and patient’s compliance [6]. Comorbidities, such as Crohn's disease (CD), may also affect warfarin pharmacokinetics [7]. Although indispensable for warfarin dosing, these factors are rarely sufficient for health caregivers to determine the optimal warfarin dose and may constitute the tip of the iceberg [2, 6]. Responsiveness to warfarin is also influenced by the genetic makeup of each individual patient. Single Nucleotide Polymorphisms (SNPs) in the genes encoding vitamin K epoxide reductase complex 1 (VKORC1), the enzyme targeted by warfarin, and CYP2C9, the major warfarin metabolizing enzyme, have been recognized as the major genetic determinants of warfarin dosing [8].

The clinically relevant SNPs in VKORC1 or CYP2C9 are those that can enhance or decrease the anticoagulation effect of warfarin, and consequently, increases the risk of hemorrhage or the likelihood of recurrent thrombosis, respectively [9].

Warfarin exhibits its anticoagulation activity via inhibiting VKORC1, thus preventing the activation of the clotting factors II, VII, IX, and X [10]. Of the well-characterized SNPs, the -1639G > A (rs9923231) located in the promoter region of the VKORC1 gene is of utmost importance due to its impact on the gene expression and dosage requirement [11].

The CYP2C9 gene is highly polymorphic, with the most prevalent and clinically relevant variants being the CYP2C9*2 (NM_000771.3:c.430C > T, p.Arg144Cys, rs1799853) and CYP2C9*3 (NM_000771.3:c.1075A > C, p.Ile359Leu, rs1057910) [12, 13]. Both CYP2C9*2 and CYP2C9*3 decrease the enzyme activity compared to the common allele*1 by 30% and 90%, respectively [14].

Although 85 other allelic variants have been reported in the CYP2C9 gene according to the latest updates of pharm VAR, the scarcity of most of these variant alleles in the world's multi-ethnic populations has hindered their assignment clinical phenotypes and confined their functional impact assessment to mostly in vitro models [15]. Exceptionally, the relatively high frequencies of the *5, *6, *8, and *11 in African descendants have made the in vivo assessment of these alleles feasible [16, 17]. Still, over 50% of the missense variants in pharm VAR have unknown significance and little clinical attention has been paid to these infrequent CYP2C9 alleles [15]. Of these less well functionally characterized alleles, the CYP2C9*46 (rs754487195), confined to the Han Chinese population, has been only in vitro assessed and the resulting evidence supported a phenotype with impaired enzymatic activity in metabolizing substrate (i.e., phenytoin) [18].

This study is the first to report a clinical case of a young patient with Crohn's disease and ultrasonography confirmed DVT who exhibited a warfarin hyper-responsive phenotype and the heterozygous CYP2C9*1*46 genotype.

### The case presentation

A 22-year-old Syrian male presented with a red, swollen, and painful left leg. His medical history included Crohn's disease, for which he received sulfasalazine (2000 mg/daily) and azathioprine (100 mg/daily). The patient had no comorbidities nor was he on any other medications.

**Fig. 1:**
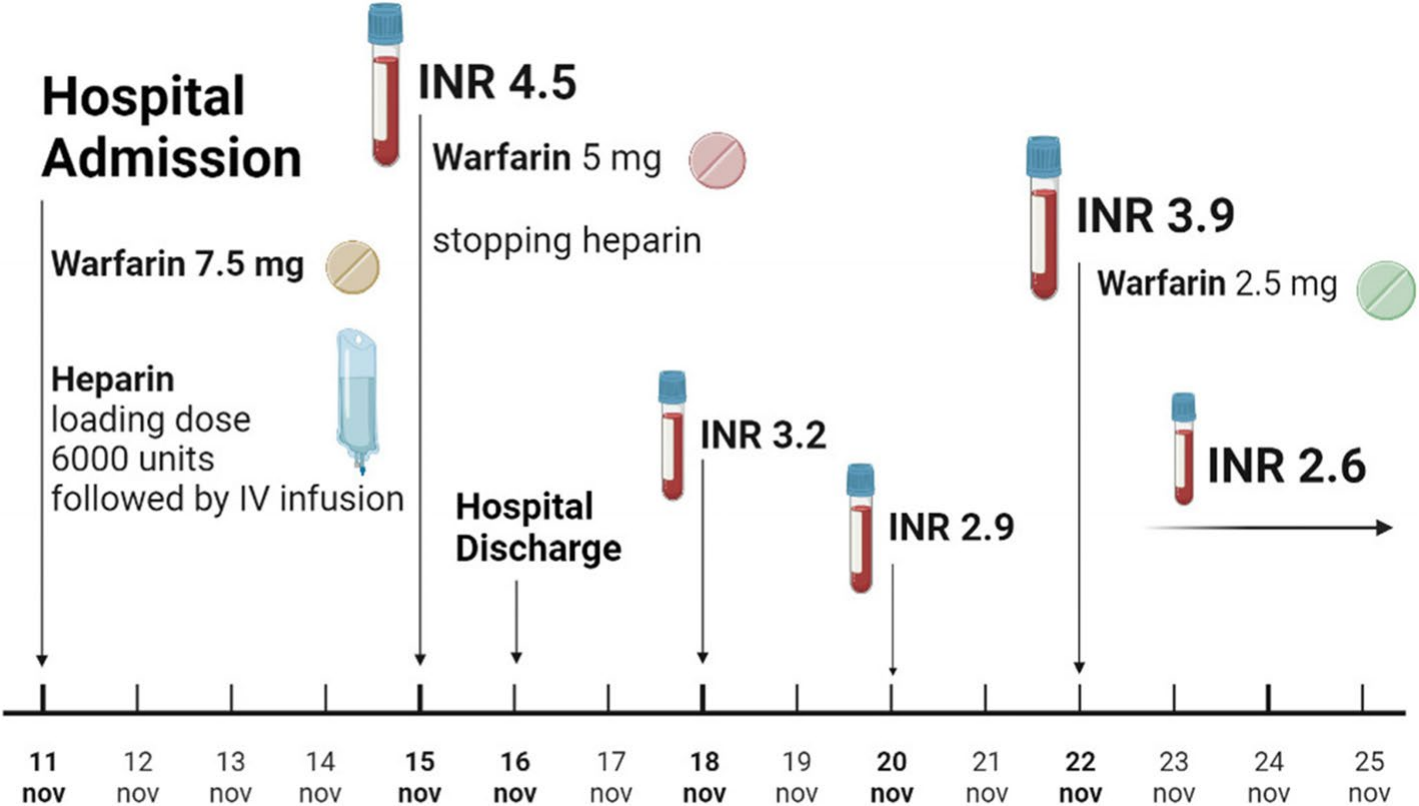
Timeline showing the case patient's warfarin dose adjustments according to INR values until reaching a stable dose. ^*^INR: International Normalized Ratio, IV: intravenous

Based on clinical examination and angiographic ultrasound, the patient was diagnosed with deep vein thrombosis in his left lower extremity, for which he was hospitalized, and a loading dose of unfractionated heparin (75 units/kg) was initiated. In accordance with his weight (80 kg, BMI = 26.1 kg/m^2^), the total loading dose of 6000 units of heparin was given as a bolus, followed by a continuous intravenous infusion of 18 units/kg/hour. Owing to his young age, being overweight and the enhancing effect of azathioprine on warfarin metabolism, the hospitalist decided to concomitantly commence warfarin at 7.5 mg daily, and INR testing was performed every other day. On the fifth day, the value of INR dramatically increased to 4.5, which obliged a reduction of warfarin dose to 5 mg daily and a halt in heparin infusion. The patient was discharged on the sixth day after admission. The next INR values were 3.2 and 2.9 on the eighth and tenth days. Then, it reached a value of 3.9 after one week of discharge. Therefore, the warfarin dose was decreased to 2.5 mg per day (30% of the initiation dose), which led to reaching and maintaining a therapeutic INR value of 2.6, as shown in Fig. (1).

**Fig. 2:**
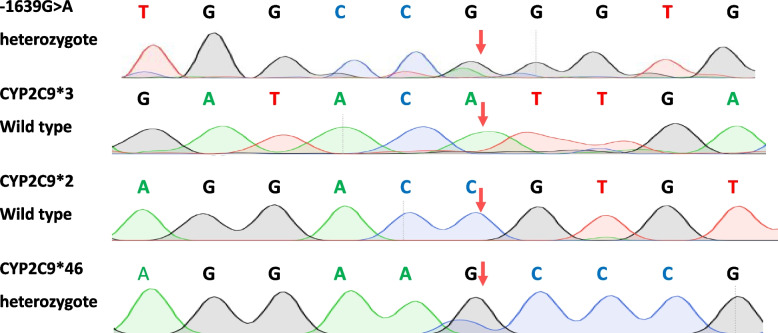
Sequencing chromatograms for (CYP2C9*2, CYP2C9*3, CYP2C9*46 & -1639G > A) SNPs

Since PGx testing has not been adapted to guide warfarin dosing in the Syrian health system, we sought to perform PGx testing for warfarin's relevant genes (i.e., CYP2C9 and VKORC1). After patient consenting, three milliliters of peripheral blood were withdrawn to an Ethylenediaminetetraacetic acid (EDTA) tube for genotyping. Then Genomic DNA was extracted using Genomic DNA Purification with Nucleospin® Blood Quick Pure, in accordance with the manufacturer's protocol. The DNA sample was amplified by Polymerase Chain Reaction (PCR) in a final volume of 50 μL. Three reactions were needed to identify the patient’s VKORC1 **-**1639G** > **A and CYP2C9 *2, *3, *4, *5, *8, *14, *20, *24, *26, *33, *40, *41, *42, *43, *45, *46, *55, *62, *63, *66 *68, *72, *73 and *78. PCR conditions and primers used are summarized in table (1). The PCR products were sequenced in Macrogen labs (Seoul, Korea) and sequencing chromatograms were read using Geneious software and are shown in Fig. (2).

## Discussion and conclusions

Approximately 50% of the inter-individual variations in warfarin doses could be attributed to genetic polymorphisms of the CYP2C9 and VKORC1 genes, together with the patient's clinical and demographic factors [19]. Here we report a case of warfarin hyper-responsiveness, manifested by above the therapeutic range INR (i.e., a supratherapeutic INR) in response to standard warfarin doses in a young man in his twenties with a history of CD managed by sulfasalazine and azathioprine.

Patients with inflammatory bowel disease (IBD), including CD, are at two to three times higher risk for developing thrombosis. Noticeably, morbidity and mortality linked to thromboembolism are significantly higher in young IBD patients and those who are hospitalized due to a flare [20]. Moreover, patients with Crohn's disease may also have reduced small intestine absorption of drugs, including warfarin, as a result of losing the effective surface area secondary to chronic inflammation, ulcerative lesions, or resection [7, 20].

On the other hand, azathioprine, indicated in a variety of autoimmune disorders such as CD, was reported to induce warfarin resistance and therefore a dose-dependent increase of at least 2.5-fold in warfarin dose requirement with the initiation of azathioprine at 75–200 mg daily [21]. Taken together, CD per se and concomitant azathioprine are expected to increase warfarin dose requirements in our case patient. Intriguingly, our patient attained high INR at a standard dose of warfarin, which could have predisposed him to serious bleeding. Due to the failure of non-genetic factors guided warfarin dose prediction, it was rational to investigate the underlying genetic factors that may elucidate this patient's hyper-responsiveness to a standard dose of warfarin.

Based on the FDA’s label and dosing algorithms tailored to individual genetic factors, the VKORC1 –1639G > A polymorphism (the AA genotype) demands lower dose requirements of warfarin [22]. Moreover, patients with CYP2C9 *1/*3, *2/*2, *2/*3, and *3/*3 genotypes are associated with lower warfarin dose requirements accompanied by a greater tendency to experience bleeding complications with standard warfarin dosing [22]. However, genotyping analysis of our patient proved the absence of the most clinically relevant CYP2C9 reduced function alleles (namely the *2,*3, *4, *5, *8, and *14) and heterozygosity of VKORC1 –1639G > A.

Accordingly, the calculated daily warfarin dose for our patient was estimated to be 6.6 mg/day based on the Gage algorithm that takes into account the patient's environmental factors and his genotype [23, 24]. Almost identically, a daily dose of 6.7 mg was conceived based on the International Warfarin Pharmacogenetics Consortium (IWPC) algorithm advocated by the Clinical Pharmacogenetics Implementation Consortium (CPIC) [24, 25]. However, such algorithm-guided dose was found to be three times higher than the real-world dose (2.5 mg/day) needed to reach and maintain INR values within the target range.

Puzzled by the failure of the different algorithm-guided warfarin dose estimations, we sought to take advantage of the lengths of the PCR products and their inclusiveness of other alleles with unknown impact or with proven in vitro reduced activity but not established clinical phenotype.

We explored the presence of unknown impact alleles (i.e., *20, *41, *47 *62, *63, *66, *68) and those with proven in vitro reduced activity (i.e., *24, *26, *33, *42, *43, *45, *46, and *55) [26]. Of these investigated allelic variants, one copy of the CYP2C9*46 (3623G > A, Ala149Thr) was detected, which makes our patient heterozygous for this allele.

Our search for the predicted phenotype of the CYP2C9*46 allele revealed paucity of relevant data. A single study by Dai and colleagues adapted an in vitro setting approach to investigate the impact of the CYP2C9*46 allele, among others, on the functionality of the encoded enzyme [18]. Based on the differential clearance rate of diclofenac as a substrate, Dai's findings proved a relatively reduced enzymatic activity of the CYP2C9*46 allele in comparison with the wild type as well as the well-established reduced function CYP2C9*3 allele [18]. Nevertheless, no in vivo investigation nor clinical evidence has supported this in vitro finding, partially due to the rarity of this allele and/or its detection in individuals not on any of the CYP2C9 substrate drugs. Building on the findings of these in vitro comparisons, we speculate a reduced enzymatic activity of the CYP2C9*46 allele by more than 90%.

The calculated daily dose for a carrier of the CYP2C9*1*3 and VKORC1 -1639GA genotype is suggested to be 4.5 mg based on the Gage algorithm [24]. Hence, warfarin dose requirement is expected to be lower than 4.5 mg daily for patient(s) with the CYP2C9*1*46 and VKORC1 GA genotype.

This is the first report of a patient on warfarin with the CYP2C9*1*46 genotype. The CYP2C9*46 allele has not been previously reported in populations other than Han Chinese [27]. The genetic makeup of the Middle East populations, including Syrians, is thought to be similar to that of the Caucasians' rather than East Asians' [28]. However, this case of a Syrian carrier of the CYP2C9*46 allele sheds the light on the diversity of the Middle Eastern populations due to human migrations, wars, and trade, which resulted in a remarkable ethnic, cultural, and genetic diversity. The currently implemented dosing algorithms (i.e., Gage, IWPC, and CPIC) take into consideration only the well-characterized and relatively frequent allele variants that result in altered activity or inactive proteins. Nevertheless, our data suggest that more attention should be paid to subjects carrying the corresponding infrequent unknown function or in vitro predicted reduced enzymatic function variants CYP2C9 alleles when prescribing warfarin. Incorporating this and other less well-characterized alleles, and probably genes, in warfarin dosing algorithms may contribute to the enhancement of their performance in the prediction of warfarin optimal dose.

**Table 1:** PCR Conditions & Primers Sequence for VKORC1 and CYP2C9 Genotyping

polymorphism or allele	Primers sequence	Product size (bp)	Annealing TM (C°)	Cycles
-1639G > A	F: 5'-GCCAGCAGGAGAGGGAAATA-3' R: 5'-AGTTTGGACTACAGGTGCCT-3'	290	60	35
*2,*8, *14,*20,*26, *33, *40, *41, *42, *43, *45, *46, *62, *63, *72, *73, *78	F: 5'-TACAAATACAATGAAAATATCATG-3' R: 5'-CTAACAACCAGGACTCATAATG-3'	690	46	40
*3,*4, *5, *24, *55, *66,*68	F: 5'-TGCACGAGGTCCAGAGATAC-3' R: 5'-ACAAACTTACCTTGGGAATGAGA-3'	105	53	35

## Data Availability

The datasets used and/or analyzed during the current study are available from the corresponding author on reasonable request.

## Abbreviations

CD: Crohn's disease
CPIC: Clinical Pharmacogenetics Implementation Consortium
DVT: Deep venous thrombosis
EDTA: Ethylenediaminetetraacetic acid
IBD: Inflammatory bowel disease
INR: International normalized ratio
IWPC: International Warfarin Pharmacogenetics Consortium
PCR: Polymerase chain reaction
PGx: Pharmacogenetics/Pharmacogenomics
PM: Personalized medicine
SNPs: Single nucleotide polymorphisms
VKORC1: Vitamin K epoxide reductase complex 1

## References

[1] Redekop WK, Mladsi D (2013). The Faces of Personalized Medicine : A Framework for Understanding Its Meaning and Scope. Value Heal.

[2] Arwood MJ, Deng J, Drozda K, Pugach O, Nutescu EA, Schmidt S, et al. Anticoagulation endpoints with clinical implementation of warfarin pharmacogenetic dosing in a real‐world setting: A proposal for a new pharmacogenetic dosing approach. Clin Pharmacol Ther. 2017;101(5):675–83. 10.1002/cpt.558 PMC5395307 28032893

[3] Hillman MA, Wilke RA, Caldwell MD, Berg RL, Glurich I, Burmester JK (2004). Relative impact of covariates in prescribing warfarin according to CYP2C9 genotype. Pharmacogenetics.

[4] Ruzickova T, Sramek M, Kaplan V, Kumstyrova S, Lacinova Z, Jansky P (2019). Warfarin loading dose guided by pharmacogenetics is effective and safe in cardioembolic stroke patients – a randomized, prospective study. Pharmacogenomics J.

[5] Özer M, Demirci Y, Candan H, Sar S, Karalt İ, Alpan S (2013). Impact of Genetic Factors ( CYP2C9, VKORC1 and CYP4F2) on Warfarin Dose Requirement in the Turkish Population. Basic Clin Pharmacol Toxicol.

[6] Shaw K, Amstutz U, Kim RB, Lesko LJ, Turgeon J, Michaud V (2015). Clinical Practice Recommendations on Genetic Testing of CYP2C9 and VKORC1 Variants in Warfarin Therapy. Ther Drug Monit.

[7] Gellatly RM, Pharm BS (2007). Intravenous Warfarin as an Alternative for Anticoagulation. Pharmacotherapy.

[8] Shi C, Yan W, Wang G, Wang F, Li Q, Lin N (2015). Pharmacogenetics-based versus conventional dosing of warfarin: A meta-analysis of randomized controlled trials. PLoS ONE.

[9] Alrashid MH, Salem AA (2016). Association of Genetic Polymorphisms in the VKORC1 and CYP2C9 Genes with Warfarin Dosage in a Group of Kuwaiti Individuals. Mol Diagn Ther.

[10] Fereidouni M, Moossavi M, Kazemi T, Nouranihassankiade S, Asghari A (2019). Association between polymorphisms of VKORC1 and CYP2C9 genes with warfarin maintenance dose in a group of warfarin users in Birjand city Iran. J Cell Biochem.

[11] Owen R (2010). VKORC1 Pharmacogenomics Summary. Pharmacogenet Genomics.

[12] Pratt VM, Cavallari LH, Tredici L Del, Hachad H, Ji Y, Moyer AM, et al. Recommendations for Clinical CYP2C9 Genotyping Allele Selection A Joint Recommendation of the Association for Molecular Pathology and College of American Pathologists. J Mol Diagnostics. 2019;21(5):746–55. Available from: 10.1016/j.jmoldx.2019.04.003 10.1016/j.jmoldx.2019.04.003 PMC7057225 31075510

[13] Daly AK, Rettie AE, Fowler DM, Miners JO (2017). Pharmacogenomics of CYP2C9: Functional and Clinical Considerations. J Pers Med.

[14] King BP, Khan TI, Aithal GP, Kamali F, Daly AK (2004). Upstream and coding region CYP2C9 polymorphisms : correlation with warfarin dose and metabolism. Pharmacogenetics.

[15] Amorosi CJ, Chiasson MA, Mcdonald MG, Wong LH, Sitko KA, Boyle G (2021). Massively parallel characterization of CYP2C9 variant enzyme activity and abundance. Am J Hum Genet.

[16] Ndadza A, Cindi Z, Makambwa E, Chimusa E, Wonkam A, Kengne AP (2019). An African Ancestral-Specific Variant Is a Strong Predictor of Dose in Black South African Patients. OMICS.

[17] Matimba A, Del-favero J, Van BC, Masimirembwa C (2009). Novel variants of major drug- metabolising enzyme genes in diverse African populations and their predicted functional effects. Hum Genomics.

[18] Dai D, Xu R, Hu L, Wang S, Geng P, Yang J (2014). CYP2C9 polymorphism analysis in Han Chinese populations : building the largest allele frequency database. Pharmacogenomics J.

[19] Biss TT, Avery PJ, Branda LR, Chalmers EA, Williams MD, Grainger JD (2012). VKORC1 and CYP2C9 genotype and patient characteristics explain a large proportion of the variability in warfarin dose requirement among children. Blood.

[20] Cheng K, Faye AS (2020). Venous thromboembolism in inflammatory bowel disease. World J Gastroenterol.

[21] Vazquez SR, Rondina MT, Pendleton RC (2008). Azathioprine-induced warfarin resistance. Ann Pharmacother.

[22] Zhang F, Finkelstein J (2019). Inconsistency in race and ethnic classification in pharmacogenetics studies and its potential clinical implications. Pharmacogenomics Pers Med.

[23] Johnson JA, Gong L, Gage BF, Scott SA, Stein CM, Anderson JL (2011). Clinical Pharmacogenetics Implementation Consortium Guidelines for CYP2C9 and VKORC1 Genotypes and Warfarin Dosing. Clin Pharmacol Ther.

[24] http://www.warfarindosing.org/Source/DoseResults.aspx. Access on 2/5/2022

[25] Zhang EJ, Jorgensen AL, Pirmohamed M (2021). Warfarin dosing algorithms : A systematic review. Br J Clin Pharmacol.

[26] Roden DM, Mcleod HL, Relling MV, Williams MS, Mensah GA, Peterson JF (2019). Series Genomic Medicine 2 Pharmacogenomics. The Lancet.

[27] Allyn-Feuer A, Ade A, Luzum JA, Higgins GA, Athey BD (2018). The pharmacoepigenomics informatics pipeline defines a pathway of novel and known warfarin pharmacogenomics variants. Pharmacogenomics.

[28] Kaye JB, Schultz LE, Steiner HE, Kittles RA, Cavallari LH, Karnes JH (2017). Warfarin Pharmacogenomics in Diverse Populations. Pharmacotherapy.

